# Properties of GaN-based light-emitting diodes on patterned sapphire substrate coated with silver nanoparticles prepared by mask-free chemical etching

**DOI:** 10.1186/1556-276X-8-157

**Published:** 2013-04-08

**Authors:** Lung-Chien Chen, Wen-Fang Tsai

**Affiliations:** 1 Department of Electro-optical Engineering, National Taipei University of Technology, 1, Sec. 3, Chung-Hsiao E. Rd, Taipei 106, Taiwan

**Keywords:** Mask-free wet etching, Silver nanoparticle, Patterned sapphire substrate, Sapphire template

## Abstract

This study reports on the use of a template that is made of silver nanoparticles (ANPs) that are dispersed on a patterned sapphire substrate (PSS) to improve the light output power of GaN-based light-emitting diodes (LEDs). The dipping of a sapphire substrate in hot H_2_SO_4_ solution generates white reaction products that are identified as a mixture of polycrystalline aluminum sulfates. These white reaction products can act as a natural etching mask in the preparation of an ANP-coated PSS (PSS-ANP) template. The optimal annealing temperature and time, surface morphology, and optical characteristics of the PSS-ANP template were investigated. The light output power of an LED that is bonded to the PSS-ANP template is approximately double than that of an LED that is not.

## Background

Rapid advances on the many fronts in the field of GaN-based technology, including in the growth of materials, have promoted the commercialization of green and blue light-emitting diodes (LEDs) and laser diodes [1]. Sapphire has been the most extensively used substrate for GaN growth owing to its relatively low cost, chemical compatibility, and stability at high temperatures. Despite considerable progress in the field of GaN-based technology, major obstacles to the realization of the full potential of these GaN-based materials are present. One of the greatest problems is the lack of a suitable substrate material on which lattice-matched GaN films can be grown. GaN heteroepitaxial films that are grown on sapphire substrate using various growth techniques have been studied widely [1-5].

The preparation of the surface of the substrate is a critical consideration in maximizing the quality of epitaxial films. To increase the internal quantum efficiency and light extraction efficiency of GaN-based LEDs, they are fabricated on a patterned sapphire substrate (PSS) [3-6]. Air gaps between GaN and the sapphire substrate can be formed by geometrically patterning the substrate to release the internal stress that is associated with the lattice mismatch that exists at the air gap, reducing the dislocation density and improving the quality of the film. Total internal reflection easily occurs in a traditional LED, so the reflection of light therein is difficult, and some light is even absorbed by the film in the LED structure. A patterned substrate can form a light-scattering area by geometry on the substrate and increase the probability of the light leaving the LEDs inside to improve the light power [7,8]. Patterned substrates can be formed by two categories of methods - dry etching and wet etching [9]. Dry etching is a method in which a gaseous chemical etching agent is used to perform non-isotropic etching, but it is likely to destroy the surface and form defects. Wet etching uses a chemical solution to etch the surface of a semiconductor isotropically; the etching rate is a function of the temperature and concentration of the solution. Such methods typically have a very high selectivity and etching rate.

The etching process comprises two steps, which are [10] (1) the diffusion of the chemical etching solution to the surface of the material that is to be etched and (2) the reaction of the chemical etching solution with the materials. Wet etching is divided into mask-associated etching and mask-free etching [10-12]. Mask-associated etching utilizes a circular array of SiO_2_ on the surface of a sapphire substrate as an etching barrier layer. The mask-free etching process uses the by-product of the reaction between the chemical etching solution and the etched material as the etching mask. However, the use of a patterning process without an additional photolithographic step can reduce manufacturing cost.

This study concerns a silver nanoparticle (ANP)-coated PSS template (PSS-ANP). The PSS-ANP is formed by sputtering a 250-nm-thick silver thin film on the PSS with heat treatment at 300°C. The PSS-ANP is a light reflector, which increases the light output power of the GaN-based LEDs.

## Methods

**Figure 1:**
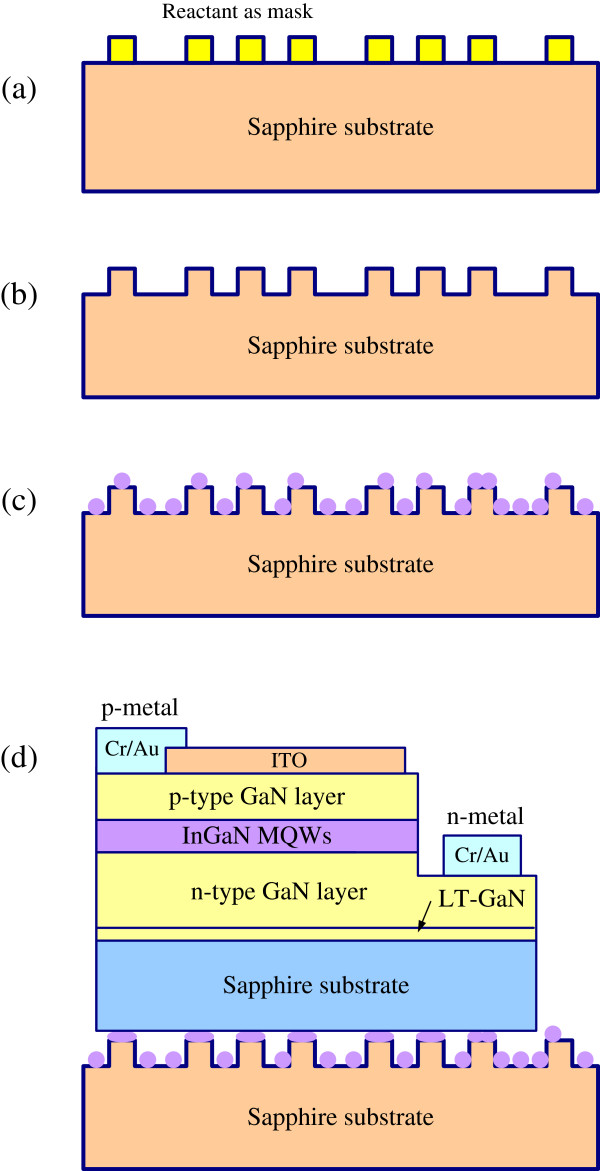
(a)-(c) preparation of PSS-ANP template and (d) cross-sectional view of complete structure.

Figure 1 presents the procedure for preparing a silver (Ag) nanoparticle-coated patterned sapphire substrate. Firstly, a chemical treatment for forming a reactant on a sapphire substrate is performed in a solution of sulfuric acid (H_2_SO_4_) at a temperature between 100°C and 250°C for 5 to 20 min. Next, the sapphire substrate is chemically etched in phosphoric acid (H_3_PO_4_) at a temperature between 100°C and 250°C for 5 to 20 min, using the reactant as an etching nanomask, to form a patterned sapphire substrate. Third, a 200-nm-thick silver film is deposited by magnetron sputtering on the patterned sapphire substrate. Finally, annealing is performed to form PSS-ANP.

Subsequently, the wafer bonding process was carried out. In this process, a GaN-based LED was directly mounted on the PSS-ANP. The LED wafer and the PSS-ANP were put together into a stainless bonding kit, which was then placed into a furnace at 500°C for 10 min. The GaN-based light-emitting diode comprised a 3-μm-thick GaN/Si layer, five pairs of undoped InGaN/GaN multiple quantum wells, and a 0.5-μm-thick layer of GaN/Mg sequentially on a (0001)-oriented patterned sapphire substrate with a GaN buffer layer that was grown by metal-organic chemical vapor deposition. Next, the surface of the p-type GaN layer was partially etched until the n-type GaN layer was exposed. A transparent conductive layer Ni/Au (50 nm:70 nm) film was formed on the p-type GaN layer. The Cr/Au (50 nm:2,000 nm) electrode was formed simultaneously on the Ni/Au film and the exposed n-type GaN layer on the front side of a wafer, respectively. Figure 1 schematically depicts the procedure for preparing the PSS-ANP template and the cross-sectional view of the complete structure. The current–voltage (*I*-*V*) and optical characteristics of LED chip on the PSS-ANP were measured.

## Results and discussion

**Figure 2:**
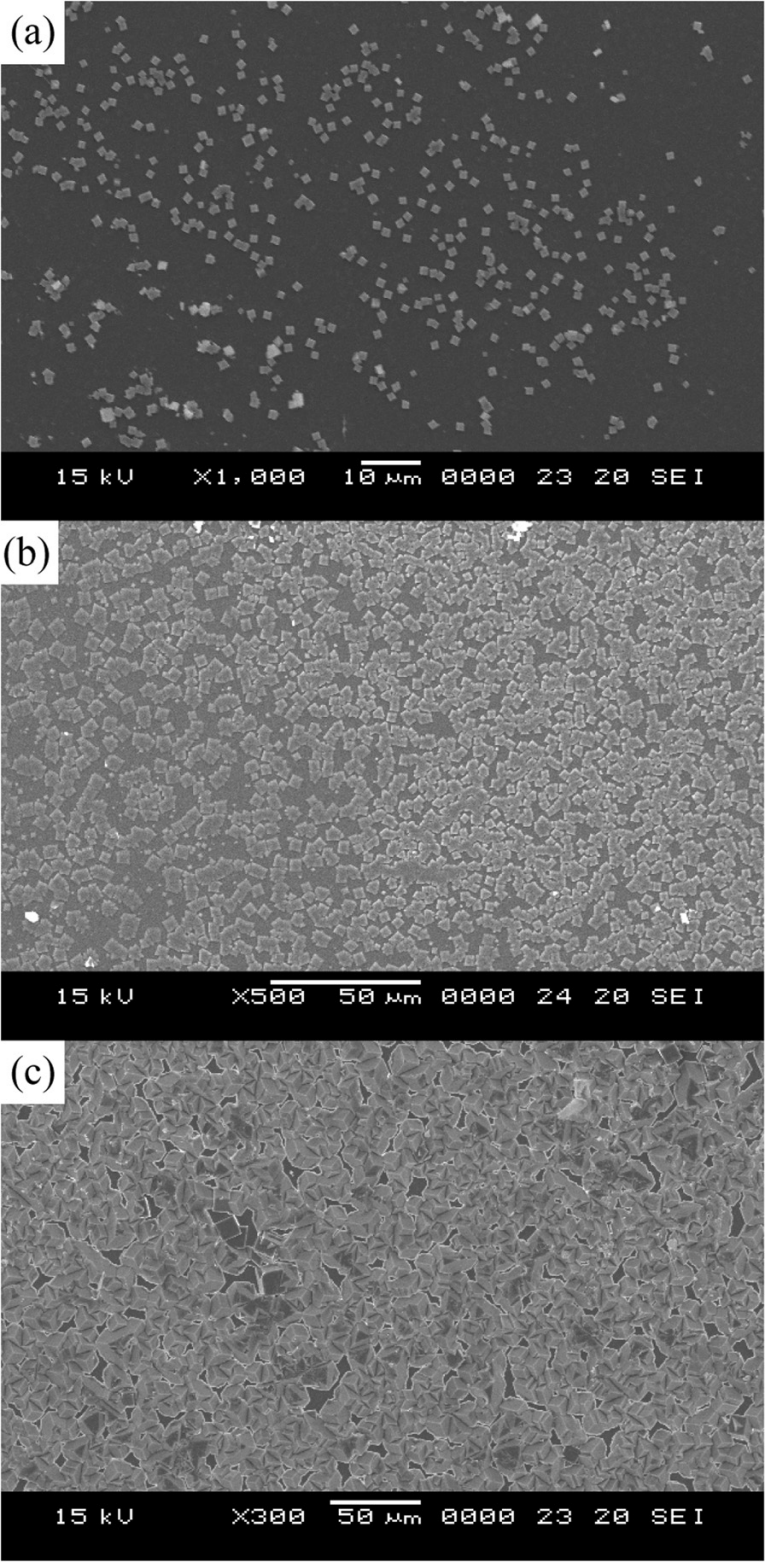
FESEM images of surface that had been etched at (a) 5, (b) 10, and (c) 20 min.

The first stages of the etching process are observed using a field emission scanning electron microscope (FESEM). Figure 2 presents top views of the sapphire substrate surface that was treated in hot sulfuric acid solution for various etching times. White lumpy crystals formed on the surfaces on the sapphire substrates during 5 min of etching (Figure 2a). The size of the lumpy crystals was approximately 1 μm. As the etching time increased, the size of the lumpy crystals increased, reaching around 10 μm after 20 min of etching. As the etching time exceeded 20 min (Figure 2c), the crystals became larger and their edges became less clearly defined, revealing the progressive dissolution of the substrate surface. The white reaction products of the sapphire substrate and the H_2_SO_4_ solution are identified as a mixture of polycrystalline aluminum sulfates, Al_2_(SO_4_)_3_ and Al_2_(SO_4_)_3_·17H_2_O [10]. These white reaction products can act as an etching mask in the subsequent etching process.

**Figure 3:**
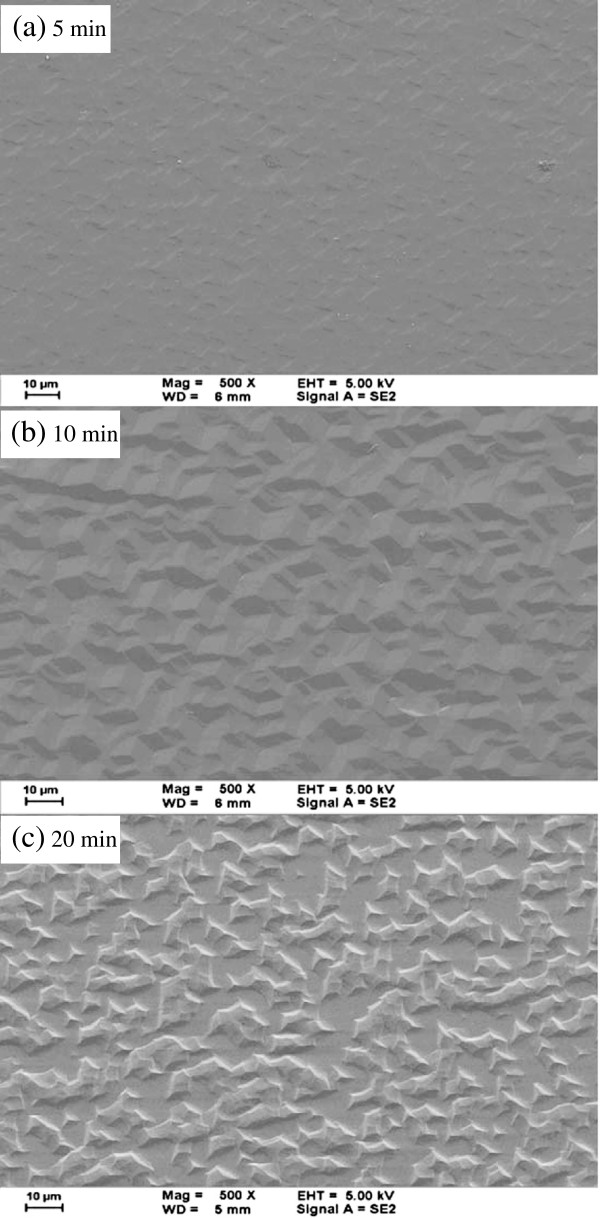
FESEM images of sapphire substrate following etching in phosphoric acid for various times.

After they had been etched in sulfuric acid, the sapphire substrates were placed in phosphoric acid at high temperature to remove the reaction products (a mixture of polycrystalline aluminum sulfates, Al_2_(SO_4_)_3_ and Al_2_(SO_4_)_3_·17H_2_O). As etching proceeded, the reaction products of size approximately 10 μm were used as a native etching mask. Figure 3 displays FESEM images of the sapphire substrates from which the reaction products on their surfaces had been cleared away to reveal terrace-like geometric patterns. As the etching time increased, the etching depth increased. At an etching time of 5 min, as shown in Figure 3a, the surface of the sapphire substrate began to exhibit the terrace-like pattern on, and the etching speed varied with the crystal plane. The etching rates of the planes of the sapphire crystalline material followed the order C-plane > R-plane > M-plane > A-plane [13]. When the sapphire was placed in hot sulfuric acid, the C-plane was the first to be etched. When the etching time exceeded 10 min, the terrace-like pattern began to appear (Figure 3b). It was formed as a combination of three R-planes. When the etching time exceeded 15 or 20 min (Figure 3c), the R-plane started to be etched, and the original terrace-like geometric patterns were destroyed.

**Figure 4:**
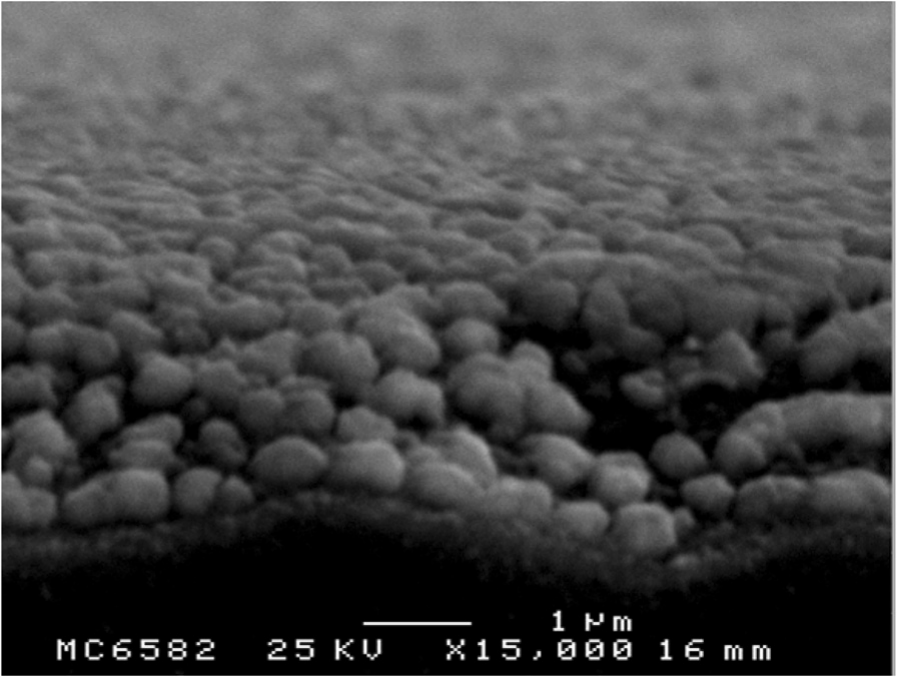
Cross-sectional FESEM image of PSS-ANP template with annealing at 500°C and etched for 5 min.

Figure 4 presents the cross-sectional FESEM image of the PSS-ANP template that had been annealed at 500°C for 5 min of etching. The silver nanoparticles were dispersed on the patterned surface of the PSS, forming the PSS-ANP template. The mean particle size was approximately 400 nm. The PSS-ANP template in the GaN-based LED structure scattered and reflected the back-emitted light from the active layer of the LED.

**Figure 5:**
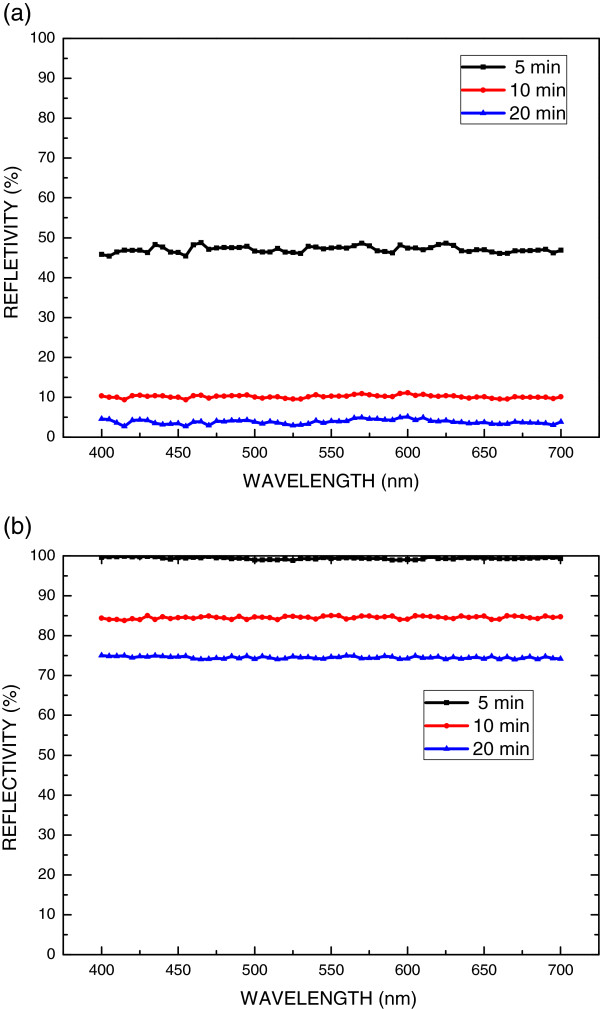
Reflectivity of (a) etched sapphire substrate and (b) PSS-ANP that had been annealed for various times.

Figure 5a plots the reflectivity of the polished sapphire substrate that had been etched for various etching times. The reflectivity of the unannealed substrate (a polished surface) was high, and it declined as the etching time increased. The integrated total reflectance from the sapphire substrate that was etched using phosphoric acid solution for 20 min was lower than approximately 5% for visible and near-infrared wavelengths. As presented in Figure 5a, the reflectance decreased as the etching time increased. The mean reflection rates in the range of 400 to 700 nm for the samples that had been etched for 5 and 20 min were 45% and 5%, respectively. As the etching time increased, the R-plane was destroyed. Figure 5b presents the reflectivity of PSS-ANP templates that had been annealed for various annealing times. The reflectivity of the PSS-ANP template that was annealed for 5 min was approximately 99.5%, which exceeded that of the PSS. This fact may have contributed to the scattering and reflection from the surface topography of the PSS-ANP.

**Figure 6:**
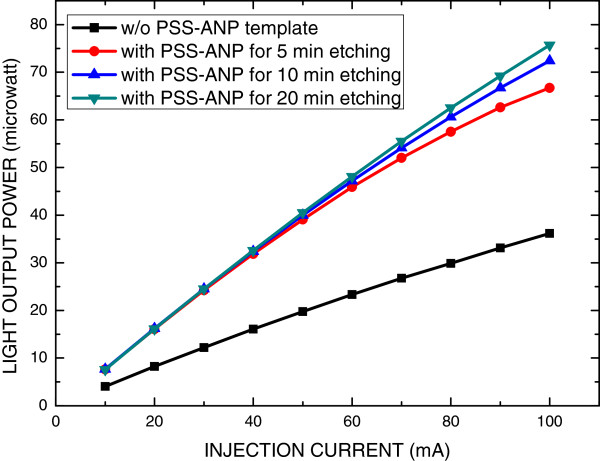
Light output power as a function of injection current of GaN LEDs with and without PSS-ANP template.

**Figure 7:**
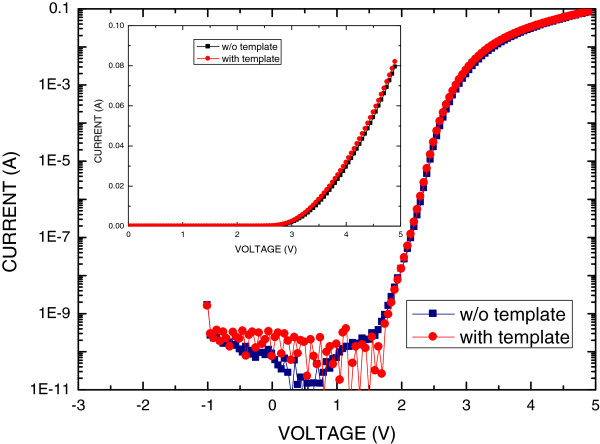
**Typical logarithmic *I*-*V *characteristics of GaN LEDs with and without the PSS-ANP template.** Inset plots *I*-*V* characteristics on linear scale.

Figure 6 plots the light output power as a function of the injection current for the GaN-based LEDs with and without the PSS-ANP template. The light output power of all of the samples initially increased linearly with the injection current. At an injection current of 20 mA, the light output power for the GaN LEDs without the PSS-ANP template was 8.24 mW. All LEDs with the PSS-ANP template had doubled the light intensity of the LED without the PSS-ANP template at a low injection current between 10 and 40 mA. However, the output intensity of LEDs with the PSS-ANP template that had been etched for 5 and 10 min was reduced as the injection current increased above 50 mA. At a high injection current, such as 100 mA, the PSS-ANP template that had been etched for 20 min doubled the light extraction. This improvement in the light output power of the LED with the PSS-ANP template that had been etched for 20 min is caused by the thermal conductive effect of the void in the template structure. Figure 7 plots the typical logarithmic *I*-*V* characteristics of the GaN LEDs with and without the PSS-ANP template. The inset plots the *I*-*V* characteristics in a linear scale. An injection current of 20 mA in the LEDs with and without the PSS-ANP template yielded forward biases of 3.7 and 3.75 V, respectively. The saturation current of both LEDs was approximately 10^−10^ A. Both LEDs had the same electrical characteristics. Accordingly, the PSS-ANP template did not influence the electrical characteristics of the GaN-based LED because the active area of the GaN-based LED with the PSS-ANP template was separate from the optical reflective area. Therefore, combining the conventional GaN-based LED with the PSS-ANP template is an excellent means of improving the light output power of a GaN-based LED on a sapphire substrate.

## Conclusion

In summary, this study reports on the construction of a template by dispersing ANPs on a PSS to improve the light output power of GaN-based LEDs. The sapphire substrate was etched in hot H_2_SO_4_ solution to produce a mixture of polycrystalline aluminum sulfates. A mixture of polycrystalline aluminum sulfates was used as a natural etching mask to prepare the PSS-ANP template. The PSS-ANP template in the GaN-based LED structure scattered and reflected the back-emitted light from the active layer of the LED. The reflectivity of the PSS-ANP template that was etched in phosphoric acid for 20 min and annealed for 5 min was approximately 99.5%. The light output power of the LED that was bonded to the PSS-ANP template was approximately double than that of the LED that was not.

## Competing interests

The authors declare that they have no competing interests.

## Authors’ contributions

LCC wrote the paper, designed the experiments, and analyzed the data. WFT prepared the samples and did all the measurements. Both authors read and approved the final manuscript.
